# Ultrasound molecular imaging for early detection of acute renal ischemia–reperfusion injury

**DOI:** 10.1002/btm2.10638

**Published:** 2024-01-03

**Authors:** Ling Ren, Yuzhuo Zhao, Tiantian Wang, Yan Tong, Ping Zhao, Fang Nie, Yukun Luo, Lianhua Zhu

**Affiliations:** ^1^ The Second Medical College of Lanzhou University Lanzhou Gansu China; ^2^ Department of Ultrasound First Medical Center of Chinese PLA General Hospital Beijing China; ^3^ Department of Nephrology, First Medical Center of Chinese PLA General Hospital, Nephrology Institute of the Chinese People's Liberation Army, State Key Laboratory of Kidney Diseases, National Clinical Research Center for Kidney Diseases Beijing Key Laboratory of Kidney Disease Research Beijing China

**Keywords:** acute kidney injury, inflammation, ischemia–reperfusion injury, microcirculation, ultrasound molecular imaging, vascular cell adhesion molecule‐1

## Abstract

**Background:**

Microcirculatory perfusion disorder and inflammatory response are critical links in acute kidney injury (AKI). We aim to construct anti‐vascular cell adhesion molecule‐1(VCAM‐1) targeted microbubbles (TM) to monitor renal microcirculatory perfusion and inflammatory response.

**Methods:**

TM carrying VCAM‐1 polypeptide was constructed by biological coupling. The binding ability of TM to human umbilical vein endothelial cells (HUVECs) was detected. Bilateral renal ischemia–reperfusion injury (IRI) models of mice were established to evaluate microcirculatory perfusion and inflammatory response using TM. Thirty‐six mice were randomly divided into six groups according to the different reperfusion time (0.5, 2, 6, 12, and 24 h) and sham‐operated group (Sham group). The correlation of TM imaging with serum and histopathological biomarkers was investigated.

**Results:**

TM has advantages such as uniform distribution, regular shape, high stability, and good biosafety. TM could bind specifically to VCAM‐1 molecule expressed by tumor necrosis factor‐alpha (TNF‐α)‐treated HUVECs. In the renal IRI‐AKI model, the area under the curve (AUC) of TM significantly decreased both in the renal cortical and medullary after 2 h of reperfusion compared with the Sham group (*p* < 0.05). Normalized intensity difference (NID) of TM at different reperfusion time was all higher than that of blank microbubbles (BM) and the Sham group (*p* < 0.05). Ultrasound molecular imaging of TM could detect AKI early before commonly used renal function markers, histopathological biomarkers, and BM imaging. AUC of TM was negatively correlated with serum creatinine (Scr), blood urea nitrogen (BUN), and Cystatin C (Cys‐C) levels, and NID of TM was linearly correlated with VCAM‐1, TNF‐α, and interleukin‐6 (IL‐6) expression (*p* < 0.05).

**Conclusions:**

Ultrasound molecular imaging based on TM carrying VCAM‐1 polypeptide can accurately evaluate the changes in renal microcirculatory perfusion and inflammatory response, which might be a promising modality for early diagnosis of AKI.

**Translational Impact Statements**

Ultrasound molecular imaging based on TM carrying anti‐VCAM‐1 polypeptide can demonstrate the change of microcirculatory perfusion and inflammatory response in AKI early and accurately. It can assess AKI earlier than commonly used renal function, biomarkers, and histopathological indicators. Ultrasound molecular imaging based on TM carrying anti‐VCAM‐1 polypeptide can provide a sensitive and specific technique to achieve early warning of AKI, thus guiding the treatment of AKI promptly and improving its prognosis.

## 1 INTRODUCTION

Acute kidney injury (AKI) is a clinical syndrome characterized by a rapid decline in renal function over a short period.^1^ AKI is characterized by high morbidity, rapid progression, and high mortality. Early detection and treatment of AKI could significantly reduce mortality and improve the prognosis.^2^, ^3^, ^4^ In clinical practice, urine output and serum creatinine (Scr) are the commonly used indicators for detecting AKI. However, urine output is easily affected by diuretics and urinary tract diseases. Scr only increases significantly when the glomerular filtration rate is below 50%, which is also susceptible to age, gender, protein intake, and medication.^5^, ^6^, ^7^ Therefore, urine output or Scr cannot accurately detect AKI at the early stage, leading to missing optimal treatment opportunities and irreversible kidney damage. Therefore, there is an urgent need for reliable and accurate early detection means for AKI.

Ischemia–reperfusion injury (IRI) is one of the most common causes of AKI. The overproduction of reactive oxygen species (ROS), hemodynamic disorders, and inflammatory responses are the primary causes of IRI‐AKI.^8^, ^9^ Numerous studies have confirmed that changes in renal microcirculatory perfusion and inflammatory response are the early manifestations of AKI before the change of serum biomarkers, such as Scr and blood urea nitrogen (BUN).^10^, ^11^ Therefore, early and accurate assessment of renal microcirculatory perfusion disorders and inflammatory response is crucial for improving renal function in AKI. Contrast‐enhanced computed tomography (CT)/ magnetic resonance imaging (MRI) has made some progress in evaluating renal perfusion and inflammatory response. Still, its clinical application is limited due to the nephrotoxicity of contrast agents.

Ultrasound molecular imaging has been widely used in the study of tumors, thrombosis, and atherosclerosis, as it can be enhanced at the molecular level by targeted ultrasound contrast agents that specifically aggregate in specific tissues to reflect changes in molecular expression in the tissue.^12^, ^13^ Some scholars have conducted experimental studies on evaluating AKI using ultrasound molecular imaging. However, it is still in the exploratory stage, and there are few studies on the early assessment of AKI. Vascular cell adhesion molecule‐1 (VCAM‐1) is a critical molecular in the development of AKI, which promote the aggregation of white blood cells in the renal, leading to damage to the endothelial cell and obstruction of microvessels, accelerating changes in renal microcirculatory perfusion and inflammatory response. There is a high expression of VCAM‐1 in vascular endothelial cells of AKI, while normal renal tissue does not express or only in trace amounts.^14^, ^15^, ^16^ Therefore, anti‐VCAM‐1 targeted ultrasound contrast agents can achieve ultrasound molecular imaging of AKI to show changes in renal microcirculatory perfusion and inflammatory responses.

In this study, we constructed targeted microbubbles (TM) carrying anti‐VCAM‐1 polypeptide to explore their specific binding ability and target‐enhanced imaging ability during the development of AKI, investigate the changes of microcirculatory perfusion and inflammatory response in IRI‐AKI, and clarify the correlation of TM in ultrasound molecular imaging with serum and histopathological biomarkers of AKI. Overall, TM carrying anti‐VCAM‐1 polypeptide may have clinical application potential for ultrasound molecular imaging and improve the accuracy of early detection of AKI.

## 2 MATERIALS AND METHODS

### 2.1 Main reagents

Details are provided in the Data S1.

### 2.2 Preparation of TM

The 1,2‐dipalmitoyl‐sn‐glycero‐3‐phosphocholine (DPPC), 1,2‐distearoyl‐sn‐glycero‐3‐phosphoethanolamine‐N‐[biotinyl (polyethylene glycol) 2000] (DSPE‐PEG2000‐Biotin), and 1,2‐dipalmitoyl‐sn‐glycero‐3‐phosphate (DPPA) in a certain proportion were dissolved in a mixture of glycerol and phosphate‐buffered saline (PBS). The mixture was heated in a water bath at 45°C for 30 min, then transferred into a penicillin bottle, filled with perfluoropropane gas, and shaken horizontally for 30 s in an ST‐B series amalgamator (TUVRheinland, Cologne, Germany) at a speed of 4500 revolutions/min. After resting in a refrigerator at 4°C for 2 h, the sample was centrifuged in a 300 *g* centrifuge for 3 min to remove the insoluble lipid material at the bottom. The blank microbubbles (BM) were obtained. Streptavidin was added to the biotinylated microbubbles suspension at the ratio of 3 μg per 10^7^ microbubbles and incubated at 4°C for 1 h. Then 0.32 μg of biotinylated VCAM‐1 polypeptide was added and incubated at 4°C for 1 h. TM carrying anti‐VCAM‐1 polypeptide was obtained (Figure S1 shows the route pattern diagram of the preparation of TM). BM without carrying anti‐VCAM‐1 polypeptide was used as control group microbubbles. To directly verify that the biotin‐streptavidin method can stably and effectively connect biotinylated polypeptides to the surface of the TM, Fluorescein Isothiocyanate (FITC)‐modified biotinylated polypeptides and BM were bound by the same process, and the lipid membrane of TM was labeled with DiI dye. Then the fluorescence intensity of the surface of TM was observed under laser confocal scanning microscopy (LCSM) (Olympus Corporation, Kyoto, Japan).

### 2.3 Characterization and detection of TM

The concentrations of TM and BM were counted using the Hemocytometer. The particle size and zeta potential of TM and BM were measured using a dynamic light scattering analyzer (Nano‐ZS; Malvern, UK). The distribution of TM and BM was observed under an optical microscope (Olympus Corporation, Kyoto, Japan), and their morphology was observed under a transmission electron microscope (TEM) (HT7700; Hitachi, Tokyo, Japan). The FACSCelesta flow cytometry (BD Biosciences, San Jose, CA) was used to detect the binding efficiency between TM and anti‐VCAM‐1 polypeptide. The biosafety of TM was assessed by a Cell Counting Kit‐8 (CCK‐8) assay and a hemolysis assay. The prepared TM were stored in a refrigerator at 4°C, and their stability was evaluated by measuring the change in concentration and particle size at 0, 1, 2, 3, and 5 days after preparation.

### 2.4 Evaluation of the binding ability of TM in vitro

Details are provided in the Data S1.

### 2.5 In vitro imaging experiments of TM

An enhanced ultrasound imaging model was prepared using 1% agarose powder. Mindray Resona R9 color Doppler ultrasonic diagnostic instrument and L11‐3U linear array probe were used to collect the images of enhanced ultrasound imaging in vitro. After fixing the probe and adjusting to the contrast‐enhanced ultrasound (CEUS) mode (mechanical index of 0.072, the gain of 65 dB), the CEUS images of TM and BM were acquired at different concentrations (1.0 × 10^8^/ml, 5.0 × 10^7^/ml, 1.0 × 10^7^/ml, 5.0 × 10^6^/ml, and 1.0 × 10^6^/ml). The contrast intensity of TM at the concentration of 5.0 × 10^6^/ml was compared before and after high mechanical index ultrasound irradiation.

### 2.6 Establishment of animal models

All animal experiments and methods were performed in accordance with the relevant guidelines and regulations approved by the Animal Ethics Committee of the Chinese People's Liberation Army (PLA) General Hospital (No. 2022‐X18‐91). Thirty‐six male C57BL/6J mice, aged 6–8 weeks, were purchased from the Medical Experimental Animal Center of Chinese PLA General Hospital.

The mice were subjected to bilateral renal IRI through an established protocol described previously.^17^ In brief, the mice were anesthetized by intraperitoneal injection of 100 μl 1% pentobarbital sodium per 10 g of body weight. After the lateral abdominal opening, the renal hilum was separated, and the renal arteries and veins were clamped using atraumatic vascular clamps. The clips were released 35 min later, and the abdominal wall and muscles were sutured. The animals were randomly divided into five groups according to the different reperfusion time (0.5, 2, 6, 12, and 24 h). The sham‐operated group (Sham group) was not subjected to clipping treatment, and other operations were the same as other groups.

### 2.7 Ultrasound imaging of TM in the IRI‐AKI model

The Mindray Resona R9 color Doppler ultrasound diagnostic instrument and the L11‐3U line array probe were applied to perform in vivo ultrasound imaging in mice. CEUS was performed when each group of mice reached the corresponding reperfusion time. The largest coronal section of the right kidney was selected, and the probe was fixed and switched into CEUS mode (mechanical index of 0.072, the gain of 65 dB, image depth of 1.5 cm, frame rate of 12 fps). TM and BM with a concentration of 5 × 10^6^/ml were injected through the tail vein of mice in random order.

The dynamic storage and timing keys were activated simultaneously to save the vibrant images for 3 min continuously. The quantitative analysis of the CEUS image was carried out using the built‐in analysis software of the ultrasound diagnostic instrument. The regions of interest (ROI) in cortical and medullary were selected, respectively, and time‐intensity curves (TICs) were plotted to obtain the area under the curve (AUC) to analyze the microcirculatory perfusion disorder of AKI.

Inflammatory response evaluation of AKI was performed using a destruction‐complementation approach. After acquiring the first 3 min of contrast images, all microbubbles were destructed for 3 s using a “flash” with a high mechanical index of 0.503. After the destruction, ultrasound imaging was performed for 10 s to acquire signals from free‐circulating microbubbles supplemented by an external imaging plane. Quantitative analysis was performed using the built‐in analysis software. The contrast intensity before the destruction pulse was the total contrast signal from target‐bound and free‐circulating microbubbles. In contrast, the contrast intensity after the destruction was from re‐perfused free‐circulating microbubbles only.^18^, ^19^ The quantification of the targeting contrast signal was calculated as the normalized intensity differences (NIDs [%]) = (pre‐destruction contrast intensity − post‐destruction contrast intensity)/pre‐destruction contrast intensity × 100%].^20^

### 2.8 Renal function and serum biomarkers of AKI

After the CEUS examination, blood was taken from the inferior vena cava of the mice. Scr, BUN, and Cystatin C (Cys‐C) levels were measured using the biochemical analyzer Hitachi‐8000 (Hitachi, Tokyo, Japan). The levels of kidney injury molecule‐1 (KIM‐1) and neutrophil gelatinase‐associated lipocalin (NGAL) were measured using enzyme‐linked immunosorbent assay (ELISA) kits.

### 2.9 Renal histopathology evaluation

The kidney was treated with 4% paraformaldehyde fixation, dehydration, and paraffin embedding, and the tissue was sectioned and stained with periodic acid‐Schiff (PAS). Ten slices with 400‐fold non‐overlapping visual fields were randomly selected, including five in the cortical region and five in the corticomedullary junction. Acute renal tubular injury score was performed blind to evaluate the loss of brushy margins, tubular dilatation, cast formation, tubular necrosis, neutrophil infiltration, etc. The tubular injury was classified into six grades^21^: 0, normal; 1 score, mild injury (involving 0% to 10%); 2 scores, moderate damage (11%–25% involvement); 3 scores, severe injury (26%–49% involvement); 4 scores, highly severe injury (50%–75% involvement); 5 scores, extensive damage (>75% involvement). All assessments were carried out by two pathologists who did not know the experimental conditions.

Immunohistochemical staining of paraffin sections was used to analyze the protein expression of VCAM‐1, and CD31 staining was used to assess renal vessel density. After routine dewaxing, endogenous peroxidase was removed using 3% H_2_O_2_, and antigen repair was performed. After goat serum was blocked at room temperature for 30 min, the sections were incubated overnight at 4°C with rabbit anti‐mouse VCAM‐1 antibody (1:500 dilution) and rabbit anti‐mouse CD31 antibody (1:2000 dilution), respectively. After rinsing, the sections were incubated with horse radish peroxidase (HRP)‐labeled goat anti‐rabbit secondary antibody for 50 min at room temperature, followed by DAB development and hematoxylin re‐staining of cell nuclei. After dehydration, clearing, and neutral gum sealing, the slices were microscopically observed and comparatively analyzed for VCAM‐1 expression and vessel density in kidney tissue at different reperfusion time. Five non‐overlapping fields of view at 400× were randomly selected. The quantification was completed by two researchers unaware of the experimental conditions.

Western blot assay was used to analyze the protein expression of VCAM‐1 (detailed procedures are provided in the Data S1).

Cell apoptosis in kidney paraffin sections was detected by TUNEL staining. Five randomly selected non‐overlapping fields of view at 400× were used to count the number of apoptotic cells. The levels of tumor necrosis factor‐alpha (TNF‐α) and interleukin‐6 (IL‐6) in kidney tissue were measured using ELISA kits.

### 2.10 In vivo distribution of TM

Detailed procedures are provided in the Data S1.

### 2.11 Statistical analysis

Statistical analyses were performed using GraphPad Prism 8.0 (GraphPad Software, Inc., San Diego, CA) and SPSS 25.0 statistical software. All quantitative data are expressed as the mean ± SD. Kolmogorov–Smirnov test was used to test the data for normal distribution. Student's *t*‐test or one‐way ANOVA were used to compare and analyze all quantitative parameters. When the variance homogeneity test *p* > 0.05, Tukey's multiple comparison post‐test was used. Otherwise, Dunnett's T3 postmortem test was applied. Pearson's correlation analysis was performed between ultrasound variables and serum and histopathological parameters. *p* < 0.05 was considered a statistically significant difference.

## 3 RESULTS

### 3.1 The characterization of TM

**FIGURE 1:**
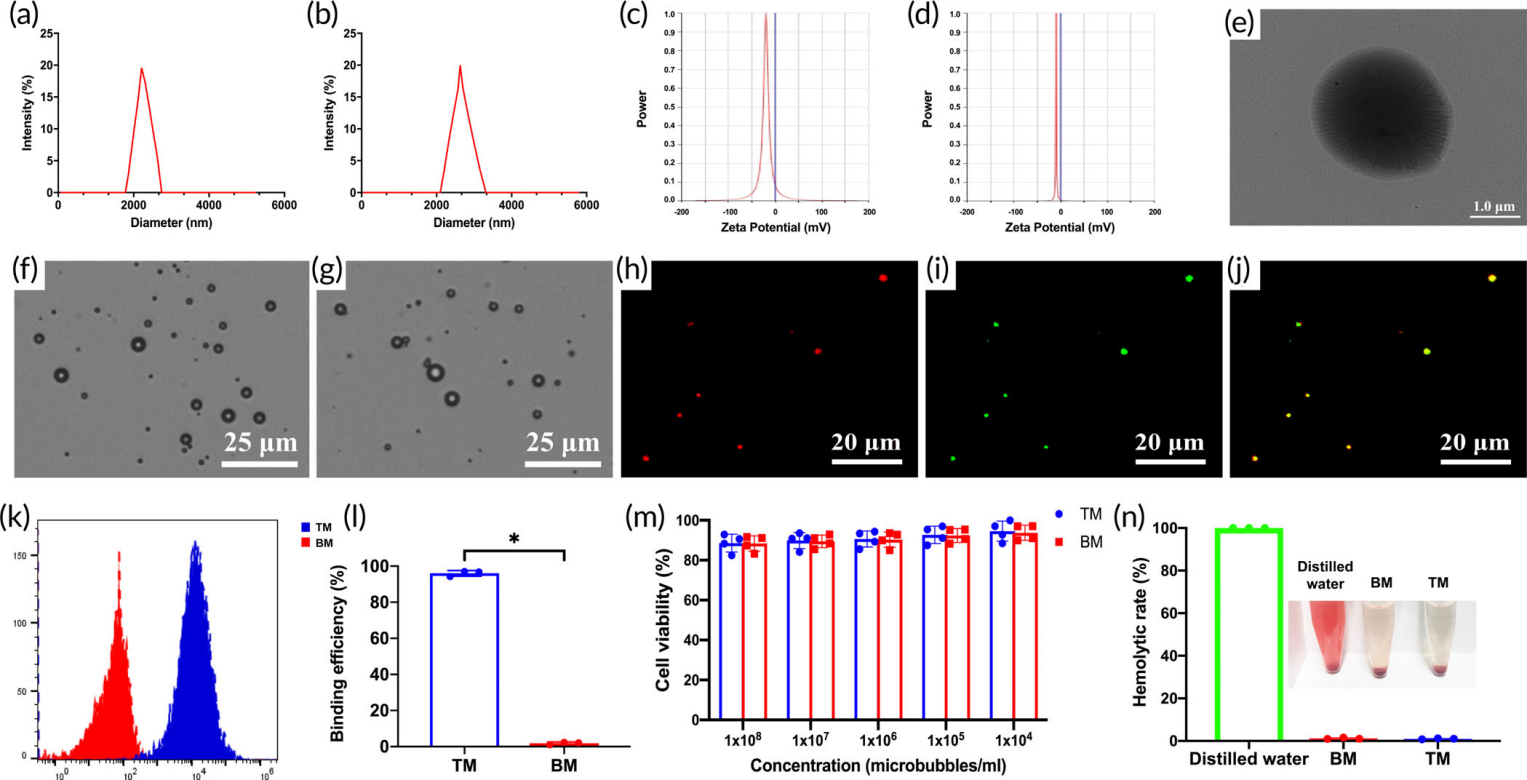
The characterization of TM. (a) The particle size of BM. (b) The particle size of TM. (c) The potential of the BM. (d) The potential of TM. (e) The morphology of TM under a transmission electron microscope. (f) The distribution of BM under an optical microscope. (g) The distribution of TM under an optical microscope. (h) DiI‐labeled lipid membrane of TM. (i) FITC‐modified polypeptides on TM. (j) The merged image confirms the polypeptides on the surfaces of TM. (k) Flow cytometry analysis of the ability of TM to carry VCAM‐1 polypeptide. (l) Quantitative analysis of the loading efficiency of TM to VCAM‐1 polypeptide (*n* = 3 per group). (m) CCK8‐assay of TM and BM (*n* = 4 per group). (n) The hemolysis experiment of TM and BM (*n* = 3 per group). Data in the graphs represent the mean ± SD, and *p* values were determined by *t*‐test or one‐way ANOVA and Tukey's post hoc test. *p < 0.05. BM, blank microbubbles; TM, targeted microbubbles.

The concentrations of BM and TM were (10.87 ± 1.10) × 10^8^/ml and (9.30 ± 0.56) × 10^8^/ml, respectively, and there was no significant difference between them (*t* = 2.199, *p* = 0.093). The detection results of the dynamic light scattering instrument showed that the particle sizes of BM and TM were (2280.23 ± 446.55) nm and (2728.61 ± 514.11) nm, respectively (Figure 1a, b). The potential of BM was (−15.72 ± 3.62) mV (Figure 1c) because of the presence of anionic phospholipid DPPA in the lipid components used to prepare BM. The potential of TM was (−7.67 ± 2.51) mV (Figure 1d), which was higher than that of BM, indicating that the positive charge of the amino group on VCAM‐1 polypeptide could neutralize part of the negative charge of anionic phospholipid, which also indirectly confirmed that TM carried anti‐VCAM‐1 polypeptide. Under the transmission electron microscope, TM were regular black circles with clear boundaries, and the particle size was about 2600 nm, consistent with the particle size measured by the dynamic light scattering instrument (Figure 1e). Under the optical microscope, BM and TM were evenly distributed and similar in particle size. There was no apparent aggregation or burst phenomenon (Figure 1f, g).

Under the laser confocal scanning microscopy (LCSM), the lipid membrane of DiI‐labeled TM showed red fluorescence (Figure 1h). FITC‐labeled anti‐VCAM‐1 polypeptide carried on the surface of TM showed green fluorescence (Figure 1i), and the two fluorescence could completely overlap and showed yellow (Figure 1j), directly confirming that anti‐VCAM‐1 polypeptide was carried on the surface of TM. The binding efficiency between TM and anti‐VCAM‐1 polypeptide detected by flow cytometry was (95.48 ± 1.98) %, significantly higher than that of BM (1.83 ± 0.74) %, and the difference was statistically significant (*p* < 0.05) (Figure 1k, l). The CCK8 experiment showed no significant cytotoxicity of BM and TM (Figure 1m). The hemolysis experiment showed that BM and TM had no significant hemolytic effect (Figure 1n).

The concentration of TM stored at 4°C decreased continuously, with concentrations of (8.23 ± 0.60) × 10^8^/ml, (6.43 ± 0.55) × 10^8^/ml, (4.40 ± 1.31) × 10^8^/ml, and (2.27 ± 1.25) × 10^8^/ml on days 1, 2, 3, and 5, respectively. After 2 days of storage, the concentration of TM was significantly lower than that of freshly prepared TM (*p* < 0.05) (Figure S2a). The particle size of TM gradually increased over time with particle sizes of (3219.30 ± 862.10) nm, (4742.04 ± 919.00) nm, (7274.72 ± 1418.09) nm, and (9074.04 ± 1546.35) nm on days 1, 2, 3, and 5, respectively. The particle size of TM stored for 3 days was significantly larger than that of freshly prepared TM (*p* < 0.05) (Figure S2b).

### 3.2 In vitro binding ability of TM

Figure S3 shows the VCAM‐1 protein expression in HUVECs under the LCSM, with nuclei in blue and coralite488‐conjugated VCAM‐1 in green. VCAM‐1 protein expression was observed in HUVECs treated with TNF‐α but not untreated cells.

**FIGURE 2:**
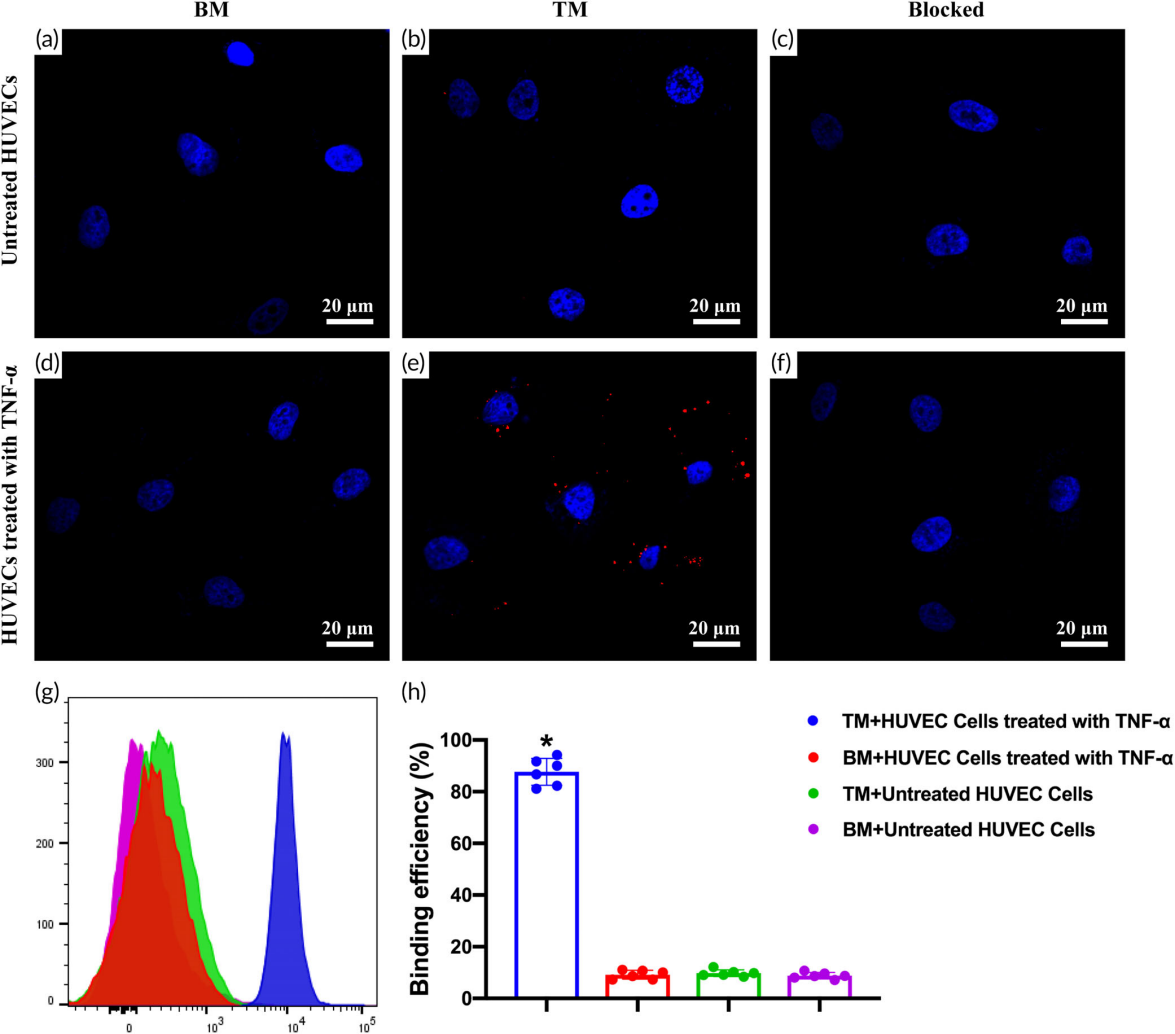
The binding ability of microbubbles in vitro. (a–f) The binding ability of microbubbles to HUVECs under laser confocal scanning microscopy. Blocked indicates the HUVECs were pre‐blocked with VCAM‐1 polypeptide. Blue fluorescence indicates cell nuclei, and red fluorescence indicates microbubbles. (g) Flow cytometry analysis of the binding ability of microbubbles to HUVECs. (h) The binding efficiency of microbubbles to HUVECs (*n* = 6 per group). Data in the graph represent the mean ± SD, and *p* value was determined by one‐way ANOVA and Dunnett's T3 post hoc test. *p < 0.05 compared with the other groups. BM, blank microbubbles; HUVEC, human umbilical vein endothelial cell; TM, targeted microbubbles; TNF‐a, tumor necrosis factor‐alpha.

The binding of microbubbles to HUVECs was observed under the LCSM. The cell nucleus was blue, and microbubbles labeled with DiI were red. The TNF‐α treated HUVECs reacted with TM, and many TM was seen around the cells, while only a tiny number of microbubbles were observed around the cells of the other groups (Figure 2a–f).

Flow cytometry was used to detect the binding ability of TM and BM to HUVECs treated with TNF‐α. The results showed that HUVECs treated with TNF‐α were significantly bound by TM. At the same time, no significant binding was observed in other groups (Figure 2g), which was consistent with the detection results of cellular immunofluorescence technology. Further quantitative analysis showed that the binding efficiency of TM to HUVECs after TNF‐α treatment was as high as (87.67 ± 5.24) %, which was significantly higher than the other groups (*p* < 0.05) (Figure 2h).

### 3.3 In vitro enhanced ultrasound imaging of TM

**FIGURE 3:**
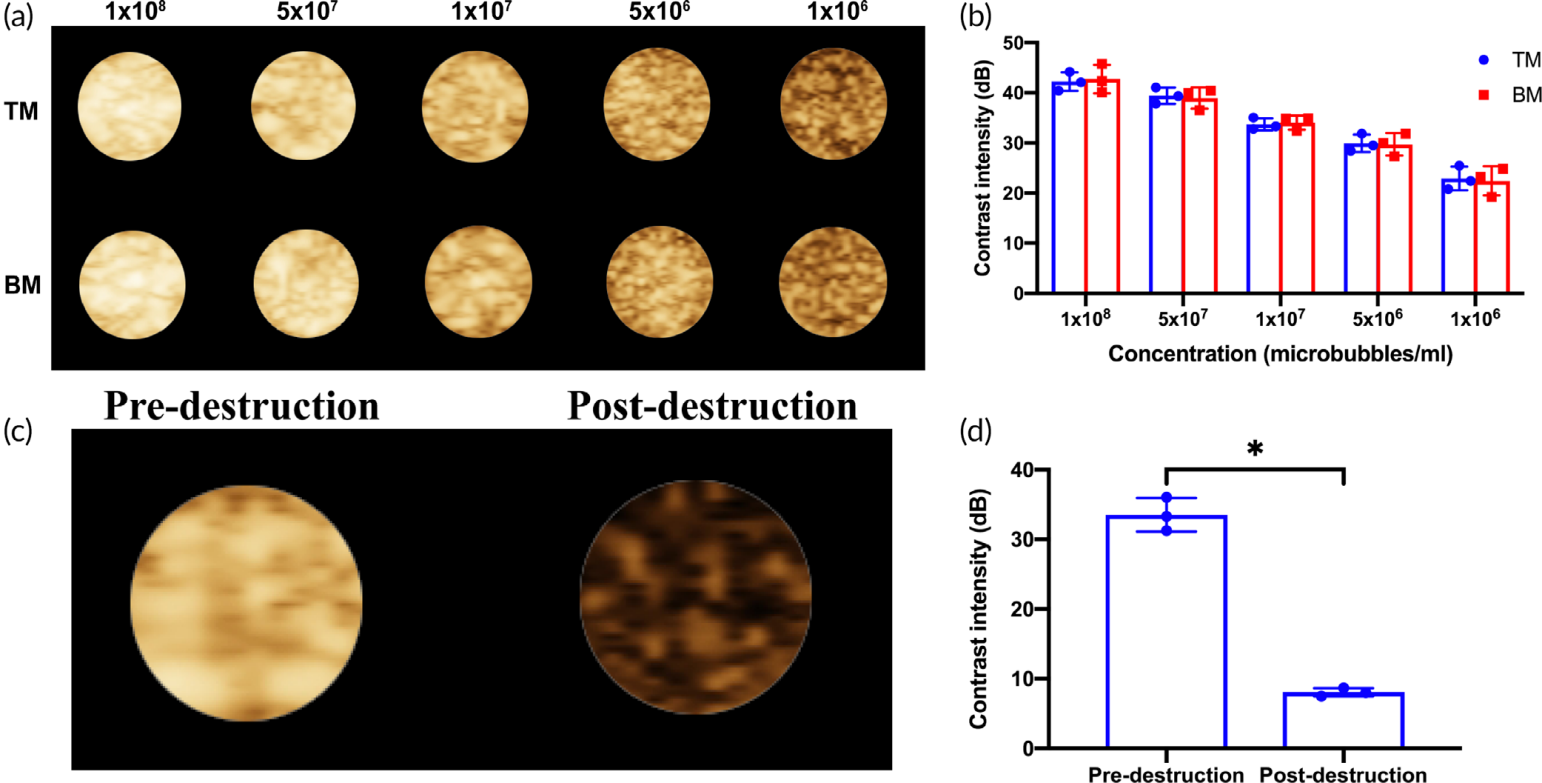
In vitro imaging of TM. (a) Ultrasound images of microbubbles at different concentrations in vitro. (b) Quantification of contrast intensity of microbubbles in vitro (*n* = 3 per group). (c) Ultrasound images of TM before and after destruction. (d) Quantification of contrast intensity of TM before and after destruction (*n* = 3 per group). Data in the graphs represent the mean ± SD, and *p* values were determined using *t* test. *p < 0.05. BM, blank microbubbles; TM, targeted microbubbles.

In vitro, the enhanced ultrasound imaging model showed that there was no statistical difference in contrast intensity of enhanced ultrasound imaging between TM and BM in each concentration gradient (*p* > 0.05) (Figure 3a, b). After high mechanical index ultrasound irradiation, the ultrasound contrast intensity significantly decreased (*p* < 0.05) (Figure 3c, d).

### 3.4 Enhanced ultrasound imaging of TM in vivo

**FIGURE 4:**
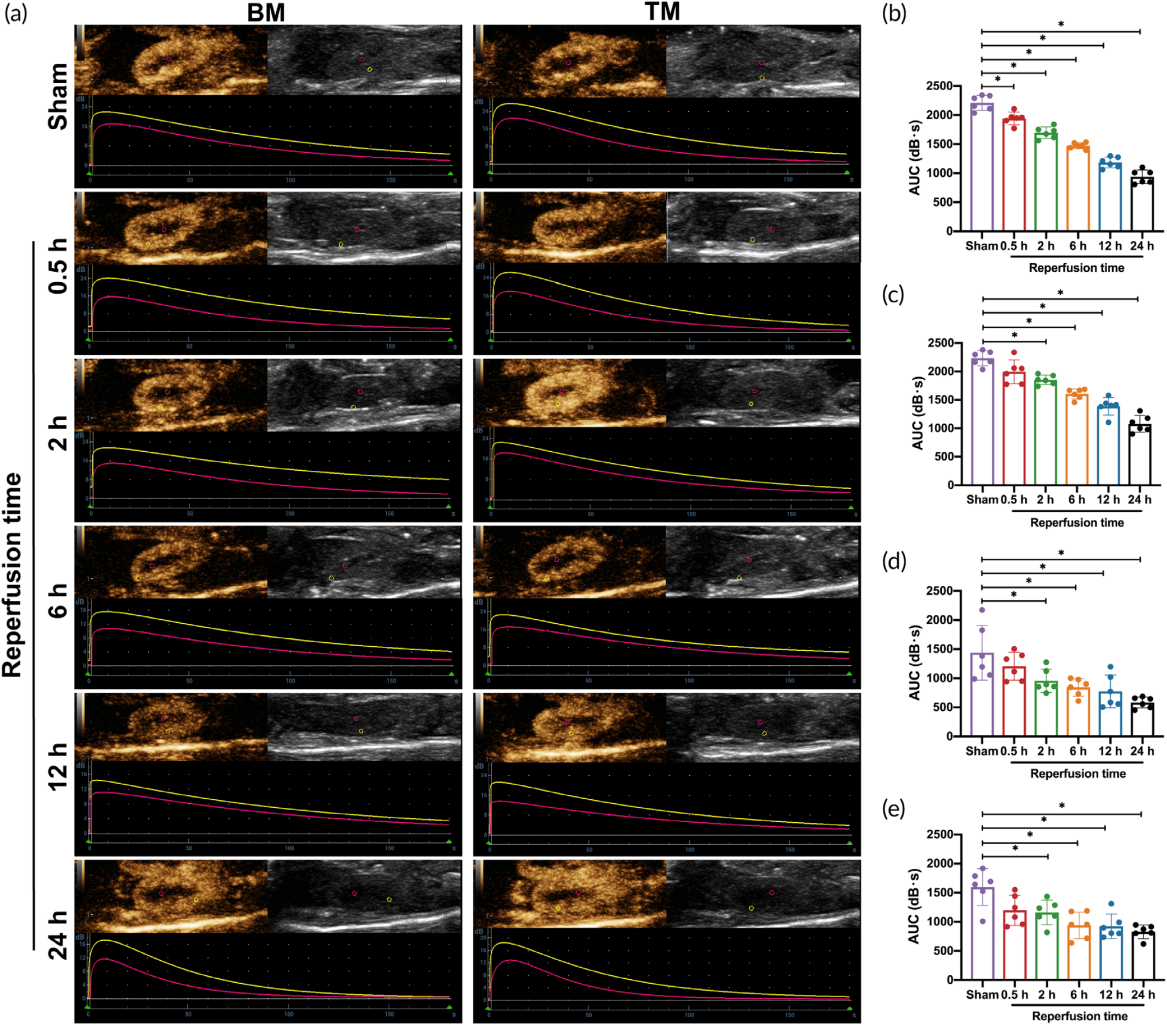
Quantitative evaluation of renal blood perfusion. (a) CEUS images for assessing renal blood perfusion by TM and BM in different reperfusion time groups and sham group. The yellow and red lines represent the time‐intensity curve of renal cortical and medullary ROI, respectively. (b) Comparison of BM in the cortical by AUC (*n* = 6 per group). (c) Comparison of TM in the cortical by AUC (*n* = 6 per group). (d) Comparison of BM in the medullary by AUC (*n* = 6 per group). (e) Comparison of TM in the medullary by AUC (*n* = 6 per group). Data in the graphs represent the mean ± SD, and *p* values were determined by one‐way ANOVA and Tukey's or Dunnett's T3 post hoc test. *p < 0.05. AUC, area under the curve; BM, blank microbubbles; TM, targeted microbubbles.

Figure 4a demonstrated the renal blood flow perfusion evaluation in the AKI mice model using TM and BM. The TICs trend of TM was consistent with that of BM during the assessment of blood flow perfusion. Both TM and BM showed a rapid peak in the cortical phase, followed by a decrease in the medullary phase. In addition, quantitative analysis results showed the cortical AUC of TM in the reperfusion group decreased with time. The cortical and medullar AUCs of TM in the reperfusion 2 h group were significantly lower than that in the Sham group, and the 24 h reperfusion group had the lowest AUC (*p* < 0.05). BM's cortical and medullary AUC trends were consistent with TM's. However, the cortical AUC of BM in the reperfusion 0.5 h group was significantly lower than that in the Sham group (*p* < 0.05) (Figure 4b–e).

**FIGURE 5:**
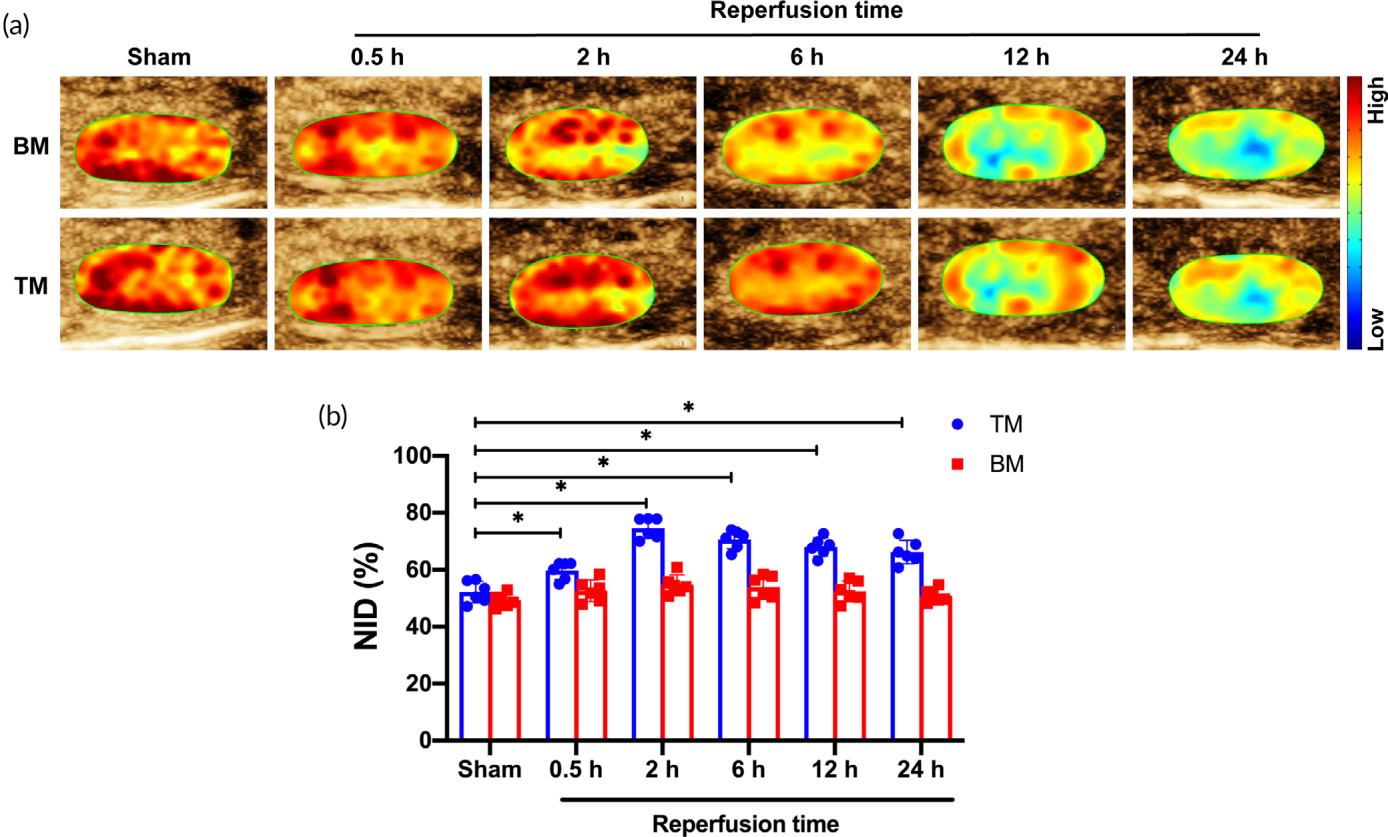
Quantitative evaluation of inflammatory response. (a) Ultrasound images for assessment of inflammatory response by TM and BM in different reperfusion time groups and sham group. (b) The comparison of NID of TM and BM (*n* = 6 per group). Data in the graphs represent the mean ± SD, and *p* values were determined by one‐way ANOVA and Tukey's post hoc test. *p < 0.05. BM, blank microbubbles; NID, normalized intensity difference; TM, targeted microbubbles.

Figure 5a showed renal inflammatory response evaluation using TM and BM in the AKI mice model. The peak intensity of TM in the reperfusion groups was higher than that of the BM. Quantitative analysis showed that compared with the Sham group, the NID of TM in all reperfusion groups was increased to varying degrees, and the highest level was found in the reperfusion 2 h group, with statistical significance (*p* < 0.05). There was no significant difference in the NID of BM between the different reperfusion time groups and the Sham group (*p* > 0.05). The Paired analysis comparison showed no statistically significant difference in the Sham group's NID between TM and BM (*p* > 0.05). In contrast, the NID of TM in the reperfusion groups was significantly higher than that of BM (*p* < 0.05) (Figure 5b).

### 3.5 Renal function and early biomarkers of AKI

**FIGURE 6:**
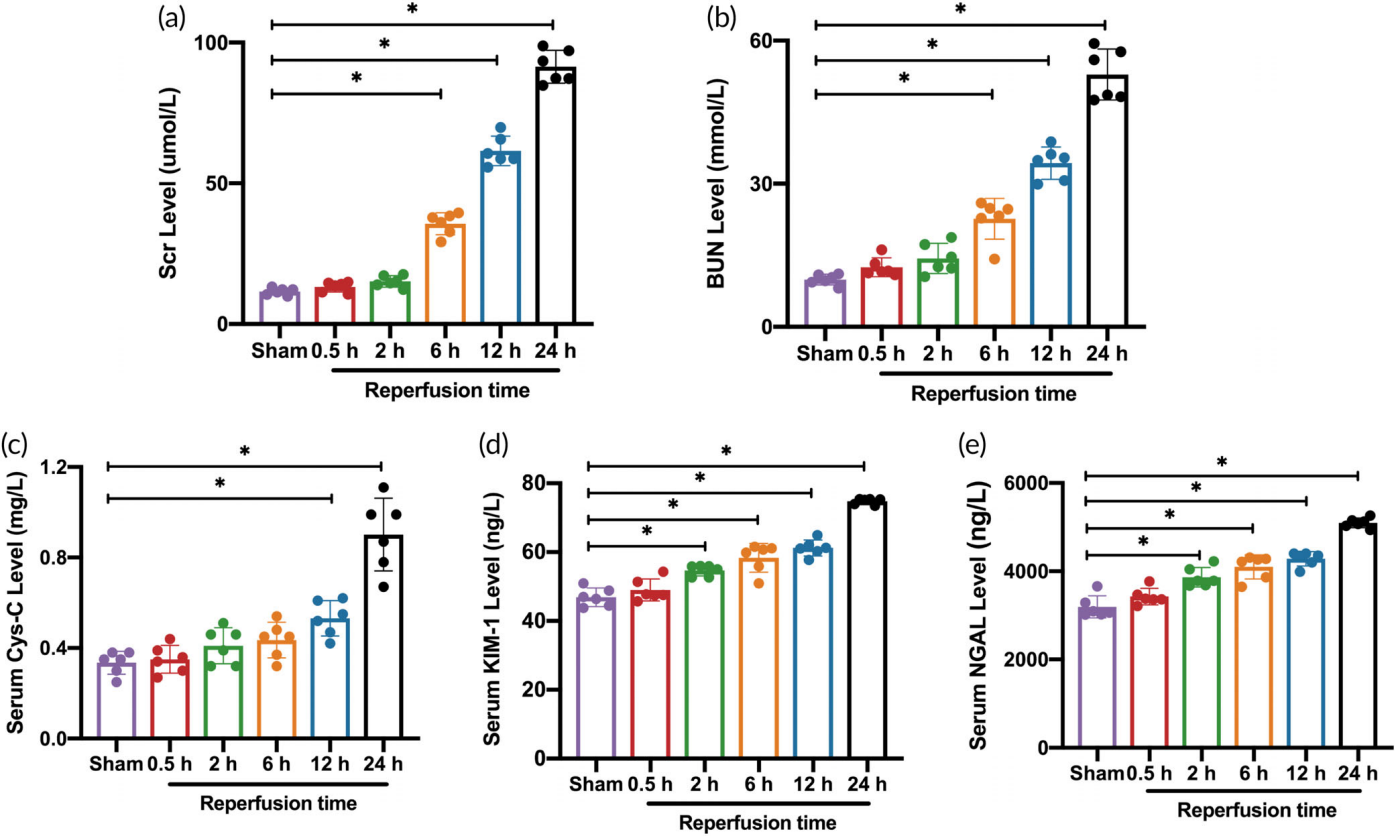
Serum biomarkers of AKI. (a) Comparison of the Scr levels (*n* = 6 per group). (b) Comparison of the BUN levels (*n* = 6 per group). (c) Comparison of the serum Cys‐C levels (*n* = 6 per group). (d) Comparison of the serum KIM‐1 levels (*n* = 6 per group). (e) Comparison of the serum NGAL levels (*n* = 6 per group). Data in the graphs represent the mean ± SD, and *p* values were determined by one‐way ANOVA and Tukey's or Dunnett's T3 post hoc test. *p < 0.05. BUN, blood urea nitrogen; Cys‐C, Cystatin C; KIM‐1, kidney injury molecule‐1; NGAL, neutrophil gelatinase‐associated lipocalin; Scr, serum creatinine.

Compared with the Sham group, the Scr and BUN levels in the reperfusion 6 h group were significantly increased, and the highest level was in the reperfusion 24 h group, with statistical significance (*p* < 0.05). There were no significant differences in Scr and BUN levels in the reperfusion 0.5 and 2 h groups compared with the Sham group (*p* > 0.05) (Figure 6a, b).

Compared with the Sham group, the Cys‐C levels in the reperfusion 12 and 24 h groups were increased, with a statistically significant difference (*p* < 0.05). The differences were not statistically significant in the reperfusion 0.5, 2, and 6 h groups compared to the Sham group (*p* > 0.05) (Figure 6c). The KIM‐1 and NGAL levels increased significantly from the reperfusion 2 h group compared to the Sham group and were highest in the reperfusion 24 h group, a statistically significant difference (*p* < 0.05). The differences in KIM‐1 and NGAL levels in the reperfusion 0.5 h group compared to the Sham group were not statistically significant (*p* > 0.05) (Figure 6d, e).

### 3.6 Renal histology evaluation

**FIGURE 7:**
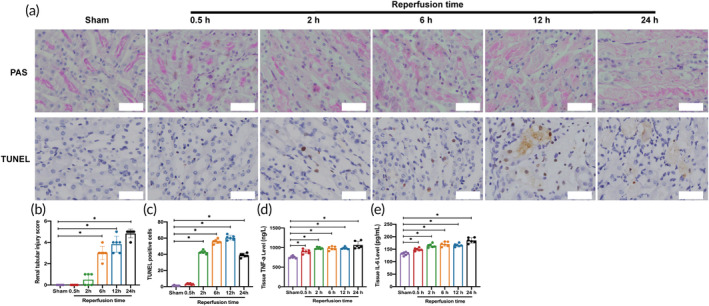
Renal histology evaluation. (a) Histopathologic images of the kidney using PAS and TUNEL staining; scale = 20 μm. (b) Comparison of the renal tubular injury score (*n* = 6 per group). (c) Comparison of the number of apoptotic cells (×400) (*n* = 6 per group). (d) Comparison of kidney tissue TNF‐a levels (*n* = 6 per group). (e) Comparison of kidney tissue IL‐6 levels (*n* = 6 per group). Data in the graphs represent the mean ± SD, and *p* values were determined by one‐way ANOVA and Tukey's or Dunnett's T3 post hoc test. *p < 0.05. IL‐6, interleukin‐6; PAS, periodic acid‐Schiff; TNF‐a, tumor necrosis factor‐alpha.

PAS staining showed that compared with the Sham group, there was no significant tubular injury in the reperfusion 0.5 and 2 h groups, and the damage was gradually aggravated in the reperfusion 6 h group. The tubular injury in the 24 h reperfusion group was the most severe, mainly at the corticomedullary junction. The renal tubules were characterized by the loss of brushy margins, tubular dilatation, and cast formation (Figure 7a). Renal tubular injury scores showed no statistically significant difference in the reperfusion 0.5 h and 2 h groups compared to the Sham group (*p* > 0.05). The difference was statistically significant in the reperfusion 6, 12, and 24 h groups compared to the Sham group (*p* < 0.05) (Figure 7b).

Figure 7a showed TUNEL staining for the detection of cell apoptosis. Compared with the Sham group, the degree of apoptosis was the least in the 0.5 h reperfusion group and gradually increased in the 2 and 6 h reperfusion groups. The apoptosis of cells reached a peak in the reperfusion 12 h group. The reperfusion 24 h group showed decreased detected cell apoptosis due to severe cell shedding. This result further indicated that the degree of AKI in the reperfusion 24 h group was the most severe. Further quantitative analysis showed that compared with the Sham group, there was no statistically significant difference in the number of apoptotic cells in the reperfusion 0.5 h group (*p* > 0.05). In contrast, the number of apoptotic cells in the other reperfusion groups was significantly higher than that in the Sham group (*p* < 0.05) (Figure 7c).

As shown in Figure 7d,e, the levels of TNF‐α and IL‐6 in renal tissue of each reperfusion group had statistical significance compared with those of the Sham group (*p* < 0.05).

### 3.7 Renal vessel density and VCAM‐1 expression

**FIGURE 8:**
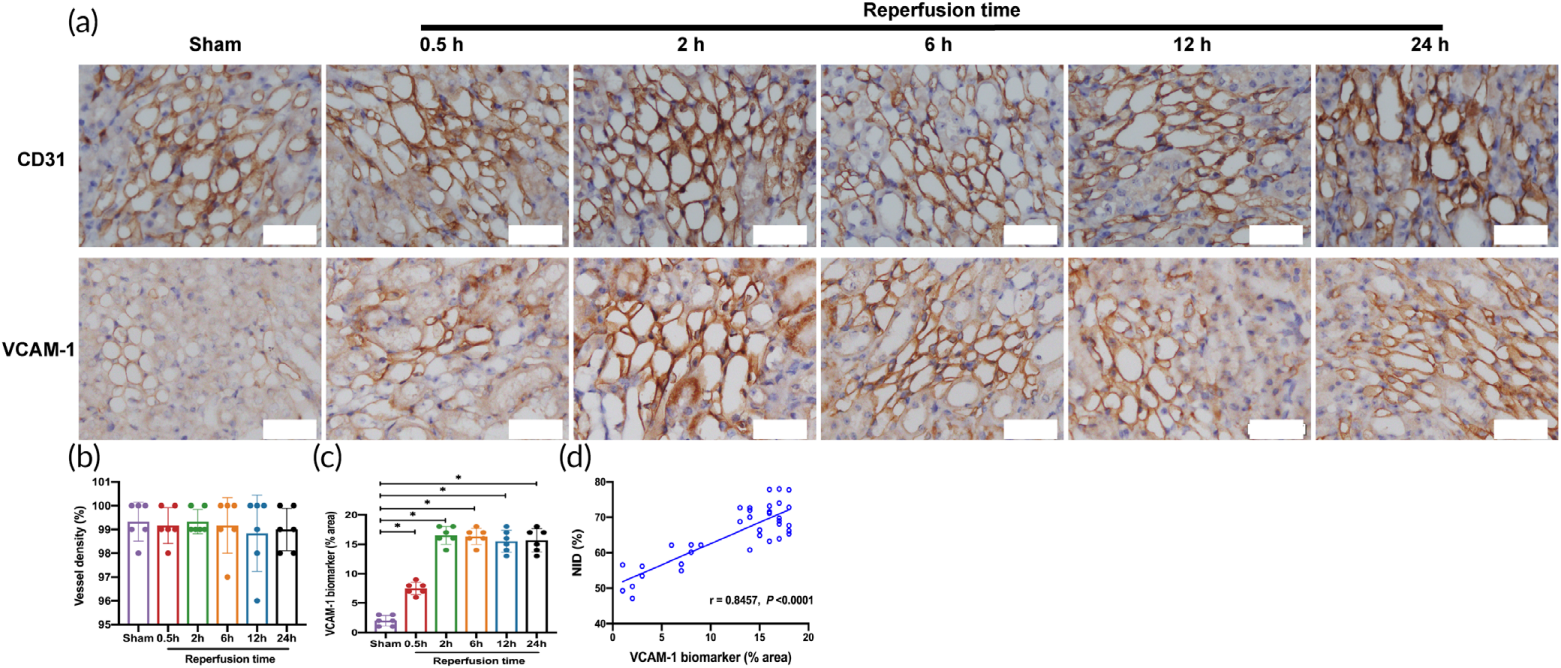
Immunohistochemical staining results. (a) Immunohistochemical image of CD31 and VCAM‐1. (b) Quantitative analysis of vessel density (*n* = 6 per group). (c) Quantitative analysis of VCAM‐1 expression (*n* = 6 per group). (d) Pearson's correlation analysis between the NID of TM and VCAM‐1 expression. Data in the graphs represent the mean ± SD, and *p* values were determined by one‐way ANOVA and Tukey's or Dunnett's T3 post hoc test. *p < 0.05. NID, normalized intensity difference; VCAM‐1, vascular cell adhesion molecule‐1.

Figures 8a and S4 show the immunohistochemical staining results of CD31 and the protein expression of VCAM‐1 in different groups. Quantitative analysis showed that there was no statistically significant difference in renal vessel density between the reperfusion groups and the Sham group (*p* > 0.05) (Figure 8b). Compared with the Sham group, VCAM‐1 expression was elevated to different degrees in all reperfusion groups, and the difference was statistically significant (*p* < 0.05) (Figures 8c and S4). In addition, Pearson's correlation analysis showed a linear positive correlation between the NID of TM and VCAM‐1 expression (r = 0.8457, *p* < 0.05) (Figure 8d).

### 3.8 Correlation analysis between ultrasound variables and serum and tissue indicators

Pearson's correlation analysis showed that NID and AUC of TM were linearly correlated with the levels of TNF‐α and IL‐6 in renal tissue of all groups (*p* < 0.05) (Figure S5a–d). In addition, the AUC of TM was linearly negatively correlated with serum Scr, BUN, and Cys‐C levels (*p* < 0.05) (Figure S5e–g). There was no significant correlation between the NID of TM and serum levels of Scr, BUN, and Cys‐C (*p* > 0.05).

### 3.9 In vivo distribution of TM

Small animal in vivo fluorescence imaging showed that after intravenous injection of DiR‐labeled TM, a large amount of aggregated fluorescent signals was observed in the kidneys of mice in the IRI group. In contrast, a small number of fluorescence signals were observed in the kidneys of mice in other groups (Figure S6a). Quantitative analysis showed that the fluorescence intensity of the kidneys in the IRI group was significantly higher than that in the other groups, and the difference was statistically significant (*p* < 0.05) (Figure S6b).

The distribution of DiR‐labeled TM in vivo was similar to that of BM. The fluorescence signal was first distributed throughout the body and then concentrated in the liver and spleen (Figure S6c,d).

## 4 DISCUSSION

In our study, TM carrying anti‐VCAM‐1 polypeptide for ultrasound molecular imaging was successfully constructed. TM had homogenous particle size, round morphology, high stability, and exemplary safety. TM can successfully bind to the VCAM‐1 molecule expressed by TNF‐α treated HUVECs through anti‐VCAM‐1 polypeptide on their surface. In the IRI‐AKI model, TM had a good enhanced ultrasound imaging effect, can accurately demonstrate the change of microcirculatory perfusion and inflammatory responses, and can be used as an agent for the early detection of AKI. Compared with common markers, including renal function markers (Scr and BUN), early biomarkers (Cys‐C, KIM‐1, and NGAL), pathological damage, and ultrasound imaging of BM, ultrasound molecular imaging based on TM carrying anti‐VCAM‐1 had higher sensitivity. Moreover, ultrasound molecular imaging based on TM carrying anti‐VCAM‐1 has good consistency with inflammatory factors (VCAM‐1, TNF‐α, and IL‐6).

In this study, we established a bilateral renal IRI model in mice. In our experiments, the presence of AKI was defined by a 1.5‐fold and higher increase in Scr according to the AKI staging proposed by the KDIGO definition.^1^, ^22^ As reported in previous literature, the bilateral renal IRI model in mice is the most stable compared with unilateral renal IRI (with or without contralateral renal resection) and is most relevant to human pathology.^17^ Regarding the selection of ischemia time, previous studies have reported that kidney ischemia time exceeding 30 min can cause severe kidney injury, and most studies have focused on ischemia time around 30–45 min. The longer the ischemia time, the more severe the kidney injury and the higher the mortality rate of the mice. We ultimately chose 35 min of ischemia to construct the AKI model to ensure the success rate.

In our study, to assess the dynamic process of AKI inflammatory response, we set up more detailed reperfusion time points to study. The reperfusion time began at 0.5 h, followed by 2, 6, and 12 h, until 24 h of reperfusion. Previous studies were limited to 4 and 24 h after reperfusion to evaluate inflammatory response.^23^ The results showed the NID of TM in different reperfusion groups increased to varying degrees, with statistically significant differences compared with the Sham group. Moreover, all the NID of TM were also higher than those of BM in the same reperfusion groups. Correlation analysis also showed that the NID of TM was positively correlated with the VCAM‐1 protein expression. In addition, studies have confirmed that TNF‐α can induce the expression of VCAM‐1 and IL‐6.^24^ Our research showed that NID of TM was linearly and positively correlated with the levels of TNF‐α and IL‐6 in renal tissues, which is consistent with previous findings. Endothelial damage and inflammatory reactions promote the occurrence and development of AKI.^5^, ^25^ Oxidative stress plays a role in all stages of AKI, and the generation of ROS activates pro‐inflammatory factors that promote renal vascular dysfunction and inflammatory responses.^4^, ^26^, ^27^, ^28^ These results indicate that ultrasound molecular imaging based on TM carrying anti‐VCAM‐1 polypeptide can effectively evaluate endothelial injury and inflammatory response in AKI in the early stage.

In the present study, we first investigated the value of TM in quantitatively assessing the microcirculatory perfusion disorder of AKI. We found that TM and BM were consistent in assessing renal microcirculation reperfusion in IRI‐AKI. The contrast intensity of both TM and BM rapidly peaked in the cortical phase, followed by a decrease in the medullary phase in AKI. Most studies have suggested that AUC has a specific value in assessing blood flow perfusion.^29^ With the severity of IRI‐AKI, renal blood perfusion becomes worse. In the cortex and medulla, both TM and BM exhibited a lower AUC in the reperfusion group than in the Sham group, consistent with the results of renal perfusion changes in canine renal IRI‐AKI assessed by Lee using SonoVue.^30^ CD31 staining showed no difference in renal vessel density in the reperfusion group compared with the Sham group, which is consistent with previous studies.^31^ The destruction of the vascular endothelial structure increases vascular permeability and intense vasoconstriction, resulting in changes in renal microcirculatory perfusion.^29^, ^32^, ^33^ In short, ultrasound molecular imaging based on TM could reflect the microcirculation disorder of AKI.

There are some limitations in our study. First, the early detection of TM in IRI‐AKI was validated only after 35 min of ischemia. Hence, further studies are needed to include different ischemia time mouse models. Second, renal IRI can cause inflammation and injury in distal organs, and we did not evaluate the impact of other organs on TM. Third, oxidative stress also plays a vital role in the occurrence and development of AKI, and future experimental research in this area will be conducted to detect AKI early from multiple perspectives. Finally, the mice model of AKI cannot fully reflect the characteristics of clinical AKI patients. Large animal models are needed to confirm the early diagnostic effect of TM for clinical translation.

## 5 CONCLUSION

In summary, TM carrying anti‐VCAM‐1 polypeptide was successfully constructed, which possessed the attractive features of high safety, high specificity, and excellent enhanced imaging effect. TM‐based ultrasound molecular imaging can early and accurately demonstrate the change of microcirculatory perfusion and inflammatory response in AKI earlier than commonly assessed markers of renal function, biomarkers, and histopathological indicators. These may provide a sensitive and specific technique to achieve early warning of AKI, thereby promptly guiding AKI treatment and improving the prognosis of AKI.

## AUTHOR CONTRIBUTIONS

**Ling Ren:** Conceptualization (equal); data curation (supporting); formal analysis (supporting); investigation (supporting); methodology (equal); writing – original draft (lead). **Yuzhuo Zhao:** Data curation (equal); formal analysis (equal); investigation (equal); methodology (equal). **Tiantian Wang:** Data curation (equal); formal analysis (equal); investigation (equal); methodology (equal). **Yan Tong:** Data curation (equal); formal analysis (equal); investigation (equal); methodology (equal). **Ping Zhao:** Data curation (supporting); formal analysis (supporting); investigation (supporting). **Fang Nie:** Supervision (equal); visualization (equal). **Yukun Luo:** Conceptualization (equal); funding acquisition (equal); investigation (equal); project administration (supporting); resources (equal); supervision (equal); visualization (equal). **Lianhua Zhu:** Conceptualization (lead); funding acquisition (lead); methodology (equal); project administration (lead); supervision (lead); validation (equal); visualization (equal); writing – review and editing (lead).

## FUNDING INFORMATION

This study was supported by the National Natural Science Foundation of China (No. 82001817), the Science and Technology “Three Talents One Team” Project of Joint Logistic Support Force of PLA (4143529).

## CONFLICT OF INTEREST STATEMENT

The authors have no relevant financial or non‐financial interests to disclose.

### PEER REVIEW

The peer review history for this article is available at https://www.webofscience.com/api/gateway/wos/peer‐review/10.1002/btm2.10638.

## ETHICS STATEMENT

The animal experiments were approved by the Animal Ethics Committee of the Chinese PLA General Hospital.

## Supporting information


**DATA S1.** Supporting Information

## Data Availability

The data that support the findings of this study are available from the corresponding author upon reasonable request.

## References

[1] Ronco C , Bellomo R , Kellum JA . Acute kidney injury. The Lancet. 2019;394(10212):1949‐1964. 10.1016/S0140-6736(19)32563-2 31777389

[2] Vijayan A . Tackling AKI: prevention, timing of dialysis and follow‐up. Nat Rev Nephrol. 2021;17(2):87‐88. 33335277 10.1038/s41581-020-00390-3 PMC7745587

[3] Birkelo BC , Pannu N , Siew ED . Overview of diagnostic criteria and epidemiology of acute kidney injury and acute kidney disease in the critically ill patient. Clin J Am Soc Nephrol. 2022;17(5):717‐735. 35292532 10.2215/CJN.14181021 PMC9269585

[4] Chen Q , Nan Y , Yang Y , et al. Nanodrugs alleviate acute kidney injury: manipulate RONS at kidney. Bioact Mater. 2023;22:141‐167. 36203963 10.1016/j.bioactmat.2022.09.021 PMC9526023

[5] Pickkers P , Darmon M , Hoste E , et al. Acute kidney injury in the critically ill: an updated review on pathophysiology and management. Intensive Care Med. 2021;47(8):835‐850. 34213593 10.1007/s00134-021-06454-7 PMC8249842

[6] Zhang WR , Parikh CR . Biomarkers of acute and chronic kidney disease. Annu Rev Physiol. 2019;81:309‐333. 30742783 10.1146/annurev-physiol-020518-114605 PMC7879424

[7] Ostermann M , Basu RK , Mehta RL . Acute kidney injury. Intensive Care Med. 2023;49(2):219‐222. 36592201 10.1007/s00134-022-06946-0

[8] Darmon M , Schnell D , Schneider A . Monitoring of renal perfusion. Intensive Care Med. 2022;48(10):1505‐1507. 36053317 10.1007/s00134-022-06857-0

[9] Moghimian M , Abtahi‐Eivary S‐H , Jajarmy N , Shahri MK , Adabi J , Shokoohi M . Comparing the effect of flaxseed and fish oils on acute ischemia‐reperfusion injury in the rat kidney. Crescent J Med Biol Sci. 2019;6(1):6‐12.

[10] Gonsalez SR , Cortes AL , Silva RCD , Lowe J , Prieto MC , Silva Lara LD . Acute kidney injury overview: from basic findings to new prevention and therapy strategies. Pharmacol Ther. 2019;200:1‐12. 30959059 10.1016/j.pharmthera.2019.04.001 PMC10134404

[11] Selby NM , Duranteau J . New imaging techniques in AKI. Curr Opin Crit Care. 2020;26(6):543‐548. 33074946 10.1097/MCC.0000000000000768

[12] Wang G , Song L , Hou X , et al. Surface‐modified GVs as nanosized contrast agents for molecular ultrasound imaging of tumor. Biomaterials. 2020;236:119803. 32028170 10.1016/j.biomaterials.2020.119803

[13] Zhang Z , Miao X , Yao W , et al. Molecular ultrasound imaging of neutrophil membrane‐derived biomimetic microbubbles for quantitative evaluation of hepatic ischemia‐reperfusion injury. Theranostics. 2021;11(14):6922‐6935. 34093862 10.7150/thno.57794 PMC8171082

[14] Mota SMB , Albuquerque P , Meneses GC , da Silva Junior GB , Martins AMC , de Francesco DE . Role of endothelial biomarkers in predicting acute kidney injury in Bothrops envenoming. Toxicol Lett. 2021;345:61‐66. 33872748 10.1016/j.toxlet.2021.04.010

[15] Yoshida T , Yamashita M , Iwai M , Hayashi M . Endothelial Kruppel‐like factor 4 mediates the protective effect of statins against ischemic AKI. J Am Soc Nephrol. 2016;27(5):1379‐1388. 26471129 10.1681/ASN.2015040460 PMC4849832

[16] Yang J , Miao X , Guan Y , et al. Microbubble functionalization with platelet membrane enables targeting and early detection of sepsis‐induced acute kidney injury. Adv Healthc Mater. 2021;10(23):e2101628. 34514740 10.1002/adhm.202101628

[17] Wei Q , Dong Z . Mouse model of ischemic acute kidney injury: technical notes and tricks. Am J Physiol Renal Physiol. 2012;303(11):F1487‐F1494. 22993069 10.1152/ajprenal.00352.2012 PMC3532486

[18] Kosareva A , Abou‐Elkacem L , Chowdhury S , Lindner JR , Kaufmann BA . Seeing the invisible‐ultrasound molecular imaging. Ultrasound Med Biol. 2020;46(3):479‐497. 31899040 10.1016/j.ultrasmedbio.2019.11.007

[19] Wang S , Hossack JA , Klibanov AL . Targeting of microbubbles: contrast agents for ultrasound molecular imaging. J Drug Target. 2018;26(5–6):420‐434. 29258335 10.1080/1061186X.2017.1419362 PMC6319889

[20] Miao X , Sha T , Zhang W , et al. Liver fibrosis assessment by viewing sinusoidal capillarization: US molecular imaging versus two‐dimensional shear‐wave elastography in rats. Radiology. 2022;304(2):473‐482. 35503015 10.1148/radiol.212325

[21] Yang B , Lan S , Dieude M , et al. Caspase‐3 is a pivotal regulator of microvascular rarefaction and renal fibrosis after ischemia‐reperfusion injury. J Am Soc Nephrol. 2018;29(7):1900‐1916. 29925521 10.1681/ASN.2017050581 PMC6050936

[22] Sparrow HG , Swan JT , Moore LW , Gaber AO , Suki WN . Disparate outcomes observed within kidney disease: improving global outcomes (KDIGO) acute kidney injury stage 1. Kidney Int. 2019;95(4):905‐913. 30819553 10.1016/j.kint.2018.11.030

[23] Hoyt K , Warram JM , Wang D , Ratnayaka S , Traylor A , Agarwal A . Molecular ultrasound imaging of tissue inflammation using an animal model of acute kidney injury. Mol Imaging Biol. 2015;17(6):786‐792. 25905474 10.1007/s11307-015-0860-6 PMC4818575

[24] Ellermann SF , Jongman RM , Luxen M , et al. Pharmacological inhibition of protein tyrosine kinases axl and fyn reduces TNF‐alpha‐induced endothelial inflammatory activation in vitro. Front Pharmacol. 2022;13:992262. 36532777 10.3389/fphar.2022.992262 PMC9750991

[25] Kellum JA , Romagnani P , Ashuntantang G , Ronco C , Zarbock A , Anders HJ . Acute kidney injury. Nat Rev Dis Primers. 2021;7(1):52. 34267223 10.1038/s41572-021-00284-z

[26] Geo HN , Murugan DD , Chik Z , et al. Renal Nano‐drug delivery for acute kidney injury: current status and future perspectives. J Control Release. 2022;343:237‐254. 35085695 10.1016/j.jconrel.2022.01.033

[27] Tafrishi Nejad S‐R , Khaki A , Abbasalizadeh S , Shokoohi M , Ainehchi N . Protective effect of Hydroalcoholic extract of Orange Peel on PCNA and FSH‐R gene expression in histological damage and oxidative stress due to ovarian torsion in adult rats. Int J Women's Health Reprod Sci. 2021;9(3):205‐211.

[28] Abadi ARR , Boukani LM , Shokoohi M , et al. The flavonoid chrysin protects against testicular apoptosis induced by torsion/detorsion in adult rats. Andrologia. 2023;2023:1‐12.

[29] Luo Z , Liu Y , Tang Z , et al. Quantitative evaluation of renal cortex perfusion using contrast‐enhanced ultrasound imaging parameters in ischemia‐reperfusion injury in rabbits. Ultrasound Med Biol. 2021;47(11):3253‐3262. 34400032 10.1016/j.ultrasmedbio.2021.07.013

[30] Lee G , Jeon S , Lee SK , et al. Quantitative evaluation of renal parenchymal perfusion using contrast‐enhanced ultrasonography in renal ischemia‐reperfusion injury in dogs. J Vet Sci. 2017;18(4):507‐514. 28385013 10.4142/jvs.2017.18.4.507 PMC5746444

[31] Zuk A , Bonventre JV . Acute kidney injury. Annu Rev Med. 2016;67:293‐307. 26768243 10.1146/annurev-med-050214-013407 PMC4845743

[32] Chen Q , Yu J , Rush BM , Stocker SD , Tan RJ , Kim K . Ultrasound super‐resolution imaging provides a noninvasive assessment of renal microvasculature changes during mouse acute kidney injury. Kidney Int. 2020;98(2):355‐365. 32600826 10.1016/j.kint.2020.02.011 PMC7387159

[33] Seo N , Oh H , Oh HJ , Chung YE . Quantitative analysis of microperfusion in contrast‐induced nephropathy using contrast‐enhanced ultrasound: an animal study. Korean J Radiol. 2021;22(5):801‐810. 33660455 10.3348/kjr.2020.0577 PMC8076825

