# Supplementary material for: Ultrasound molecular imaging for early detection of acute renal ischemia–reperfusion injury
Source: Bioeng Transl Med. 2024 Jan 3;9(4):e10638. doi: 10.1002/btm2.10638 (PMC11256142; doi:10.1002/btm2.10638)
Supplement: Supplementary file 1 — DATA S1. Supporting Information [file BTM2-9-e10638-s001.docx]

**2. Materials and Methods**

**2.1 Main reagents**

1,2-dipalmitoyl-sn-glycero-3-phosphocholine (DPPC), 1,2-dipalmitoyl-sn-glycero-3-phosphate (DPPA), and 1,2-distearoyl-sn-glycero-3-phosphoethanolamine-N- [biotinyl (polyethylene glycol) 2000] (DSPE-PEG2000-Biotin) from Xi’an ruixi Biological Technology Co., Ltd (Xi’an, China). Streptavidin from Solarbio Science & Technology Co., Ltd (Beijing, China). Polypeptide with a high affinity for VCAM-1 (VHPKQHRGGSKGC) was synthesized by Sangon Biotech (Shanghai, China). Perfluoropropane gas from Shanglan Helium Industry Technology Co., Ltd (Tianjin, China). DiI dye from Beyotime Institute of Biotechnology (Shanghai, China). Cell Counting Kit-8 (CCK-8) from Fubaike Biotechnology Co., Ltd (Beijing, China). Tumor necrosis factor-alpha (TNF-α) from PeproTech (New Jersey, USA). TdT-mediated dUTP Nick-End Labeling (TUNEL) staining from Roche (Basel, Switzerland). Rabbit anti-mouse VCAM-1 monoclonal antibody and Rabbit anti-mouse CD31 monoclonal antibody from Abcam, Coralite488-conjugated goat anti-rabbit IgG from Proteintech and goat Anti-Rabbit IgG Secondary Antibody (HRP) from Sino Biological (Beijing, China). Kidney injury molecule-1 (KIM-1), neutrophil gelatinase-associated lipocalin (NGAL), TNF-α, and interleukin-6 (IL-6) enzyme-linked immunosorbent assay (ELISA) Kits from Jiangsu Meimian Industrial Co., Ltd (Jiangsu, China).

**2.4 Evaluation of the binding ability of TM in vitro**

The human umbilical vein endothelial cells (HUVECs) cell line (purchased from ATCC, Manassas, VA, USA) was used for the study. The logarithmic growth of HUVECs was inoculated in a 12-well plate, cultured overnight, starved in serum-free medium for 24 h, and then treated with 10 ng/ml TNF-α (the cells in the untreated group were only starved). 24 h later, the cells were fixed with 4% paraformaldehyde and blocked with 1% bovine serum albumin (BSA)-PBS at room temperature. The cells were incubated with rabbit anti-human VCAM-1 antibody (1:100 dilution) overnight at 4°C, and then coralite488-conjugated goat anti-rabbit secondary antibody (1:100 dilution) was incubated at room temperature for 2 h. Cell nuclei were stained with DAPI, and VCAM-1 expression of cells was observed under the LCSM.

Untreated HUVECs and TNF-α treated HUVECs were reacted with 100 ul of DiI-labelled TM and BM at 2x10^7^/ml, respectively, while a blocking group was set up. The treated cells were pre-closed with 100 ul of anti-VCAM-1 polypeptide solution at 1 mg/ml concentration for 2 h before reacting with equal concentrations of TM. All groups were reacted at 4°C for 2h, and the nuclei were stained with DAPI. The binding of cells and microbubbles was observed under the LCSM. The FACSCelesta flow cytometry was used to analyze the binding ability of TM to HUVECs quantitatively. Untreated HUVECs and TNF-α treated HUVECs were collected into flow tubes at a concentration of 5x10^5^/tube. 500 ul of DiI-labelled TM or BM were added to each tube at a concentration of 2x10^7^/ml and incubated for 40 min at 37°C. After centrifugation, the supernatant was removed, re-suspended with 200 ul PBS, and assayed by flow cytometry.

**2.9 Renal histopathology evaluation**

Western blot assay was used to analyze the protein expression of VCAM-1. Renal tissues were lysed in RIPA buffer containing the protease inhibitor phenylmethanesulfonyl fluoride (PMSF), and the protein concentration was determined using the bicinchoninic acid (BCA) method. Total protein (50–80μg) was separated by sodium dodecyl sulfate-polyacrylamide gel electrophoresis (SDS-PAGE) and then transferred onto a nitrocellulose filter membrane, followed by incubation with primary antibodies against VCAM-1 (1:2000) overnight at 4 °C. After washing with Tris-buffered saline and Tween 20, the membrane was incubated with secondary antibody at room temperature for 60 min. The results were analyzed using ImageJ software. GAPDH was used as an internal reference.

**2.****10 In vivo distribution of TM**

To further evaluate the aggregation ability of TM in the mice kidneys, fluorescence imaging was performed on the kidneys of mice in the reperfusion 2 h and Sham groups. Mice were injected with 100ul of DiR-labelled BM and TM at a concentration of 5x10^7^/ml via the tail vein. The mice were euthanized when the microbubbles circulated in vivo for 3 min, and the kidneys were immediately collected for in vitro imaging using the small animal in vivo fluorescence imaging system (IVIS Lumina, PerkinElmer, USA). The fluorescence images of the two groups of kidneys were compared under the same fluorescence scale.

A small animal in vivo fluorescence imaging was performed on mice to evaluate the pharmacokinetics of TM in vivo. Mice were injected via the tail vein with 100 μl of DiR-labelled TM and BM at a concentration of 5x10^7^/ml. Mice were fluorescently imaged before microbubbles injection, 1 min, 3 min, 5 min, 10 min, 30 min, 1 h, 2 h, 6 h, 12 h, and 24 h after injection using a small animal in vivo fluorescence imaging system. Then the mice were euthanized, and the major organs (heart, liver, spleen, lung, and kidney) were collected for in vitro imaging to determine the distribution of microbubbles.

**Supplementary Figure Legends**

**
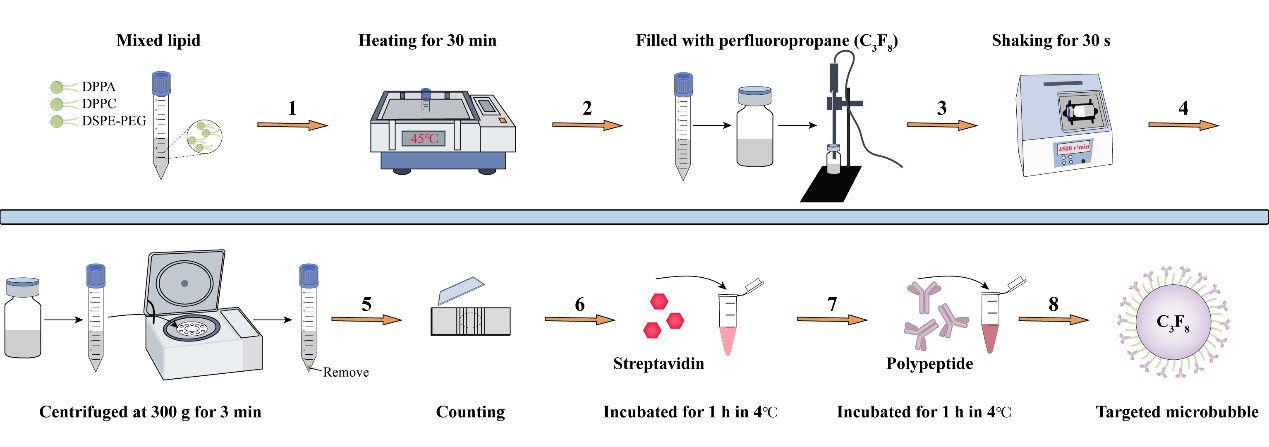
**

**Supplementary Figure 1. The route pattern diagram of the preparation of TM.**

**
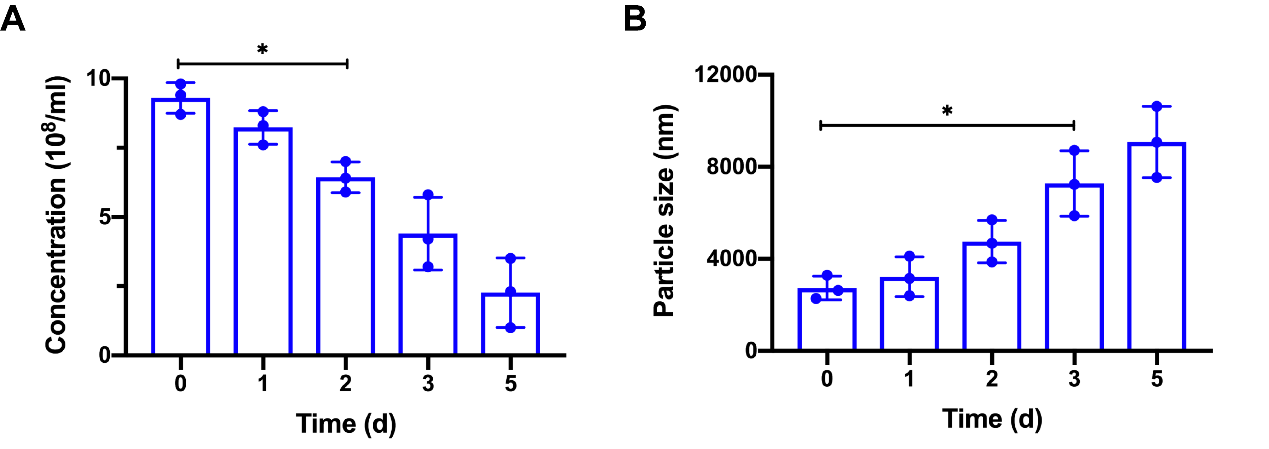
Supplementary Figure 2. Stability of TM.** (A) The concentration changes of TM. (B) The particle size changes of TM (n=3 per group). Data in the graphs represent the mean ± SD, and p values were determined by one-way ANOVA and Tukey's post hoc test. *P < 0.05.


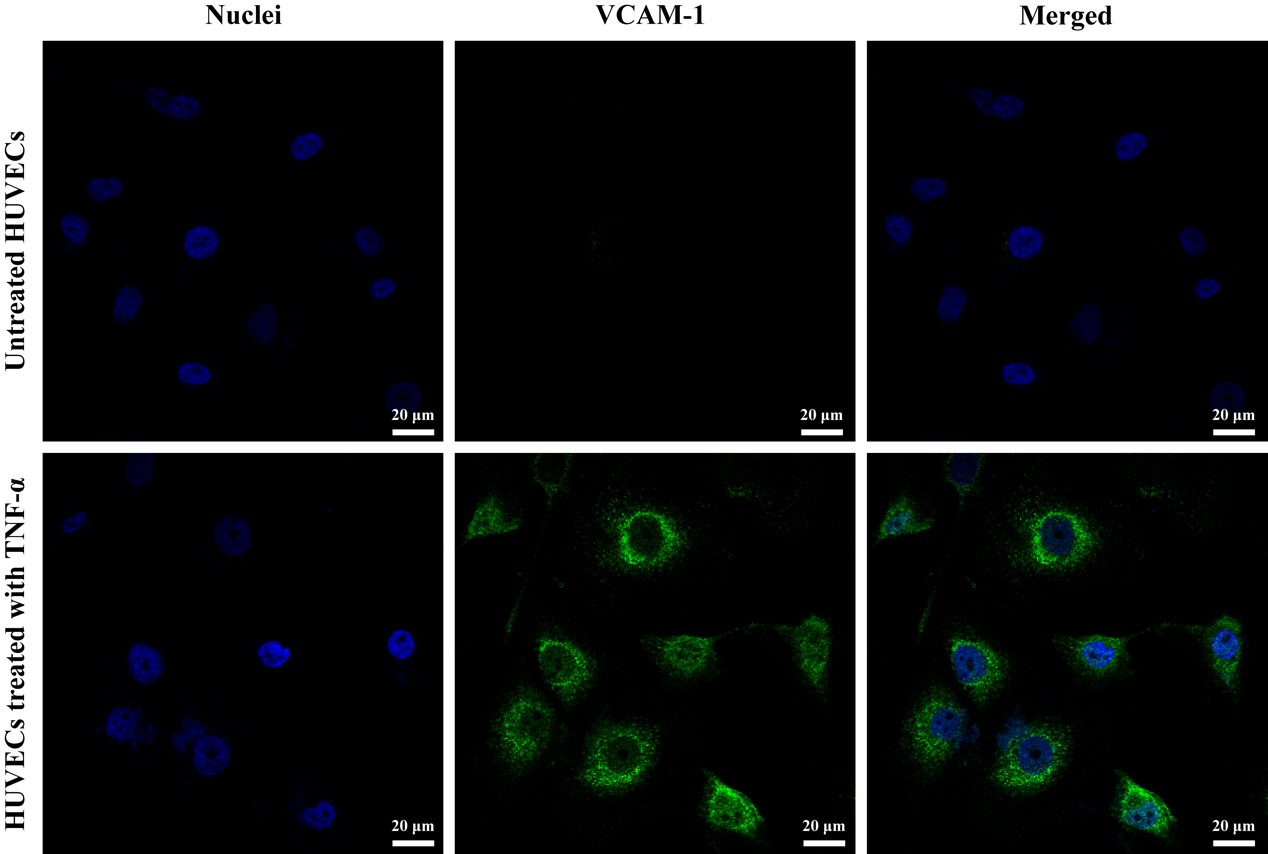


**Supplementary Figure 3. The expression of VCAM-1 in HUVECs under the laser confocal scanning microscopy.** HUVEC: human umbilical vein endothelial cell; TNF-a: tumor necrosis factor-alpha; VCAM-1: vascular cell adhesion molecule-1.

**
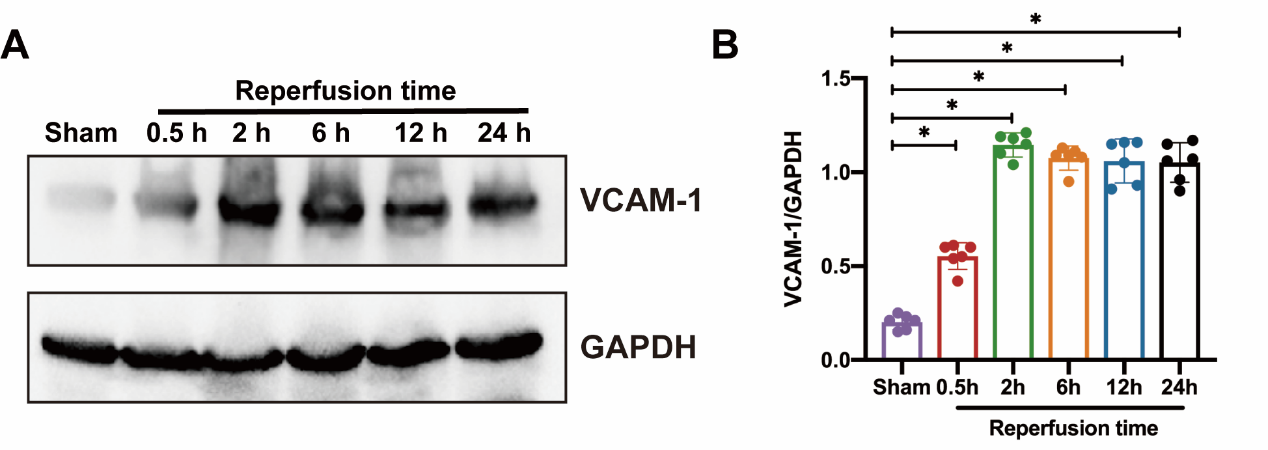
**

**Supplementary Figure 4. Renal histology evaluation.** (A) The protein expression of VCAM-1 by Western blot assay. (B) Quantitative analysis of VCAM-1 expression (n=6 per group). Data in the graphs represent the mean ± SD, and p values were determined by one-way ANOVA and Tukey's post hoc test. * P <0.05. VCAM-1: vascular cell adhesion molecule-1.


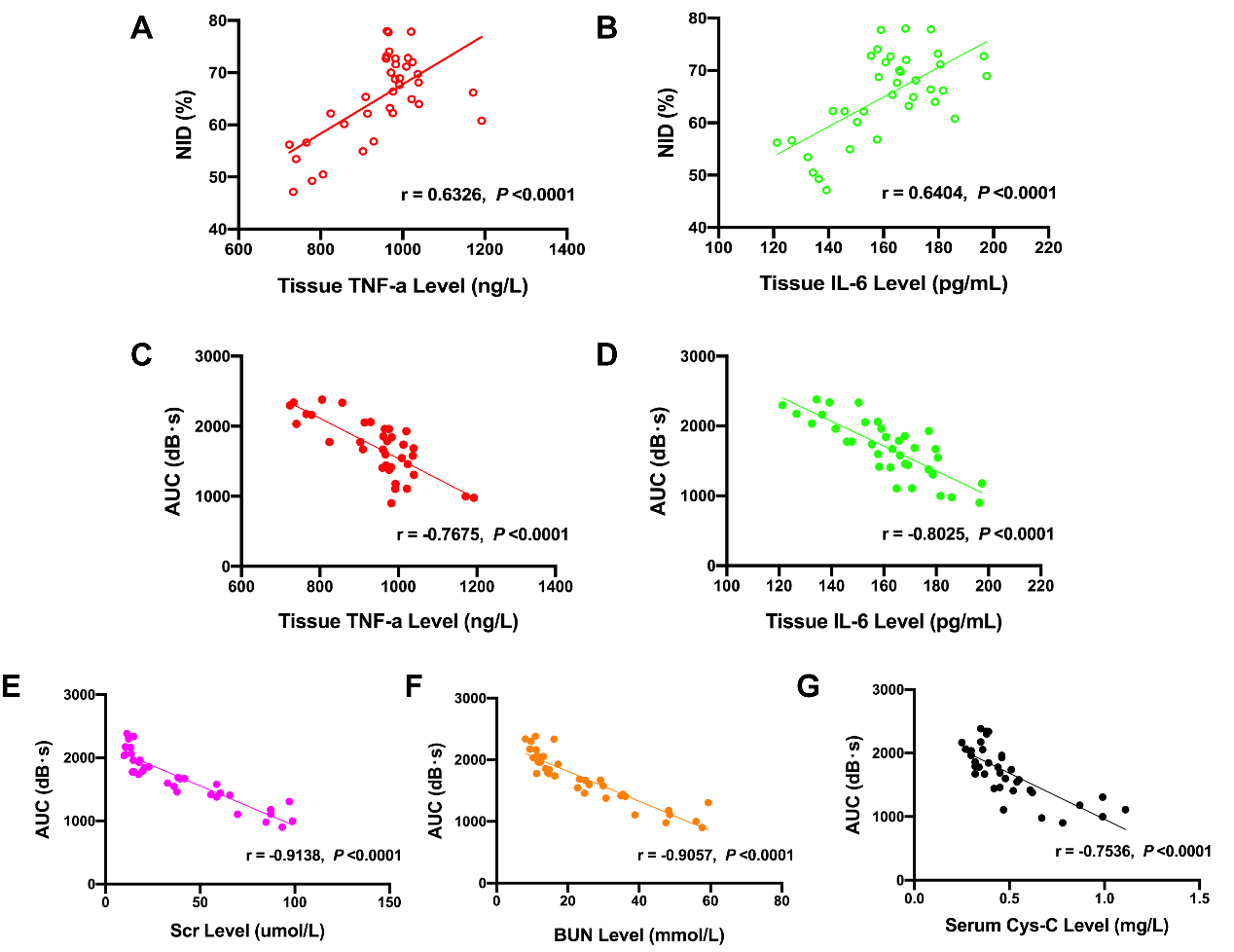


**Supplementary Figure 5. The correlation between ultrasound variables and serum and tissue indicators.** (A-B) The correlation between the NID of TM and tissue TNF-a level and tissue IL-6 level. (C-G) The correlation between the cortical AUC of TM and tissue TNF-a level, tissue IL-6 level, Scr level, BUN level, and serum Cys-C level. AUC: area under the curve; BUN: blood urea nitrogen; Cys-C: Cystatin C; IL-6: interleukin-6; NID: normalized intensity difference; Scr: serum creatinine; TNF-a: tumor necrosis factor-alpha.


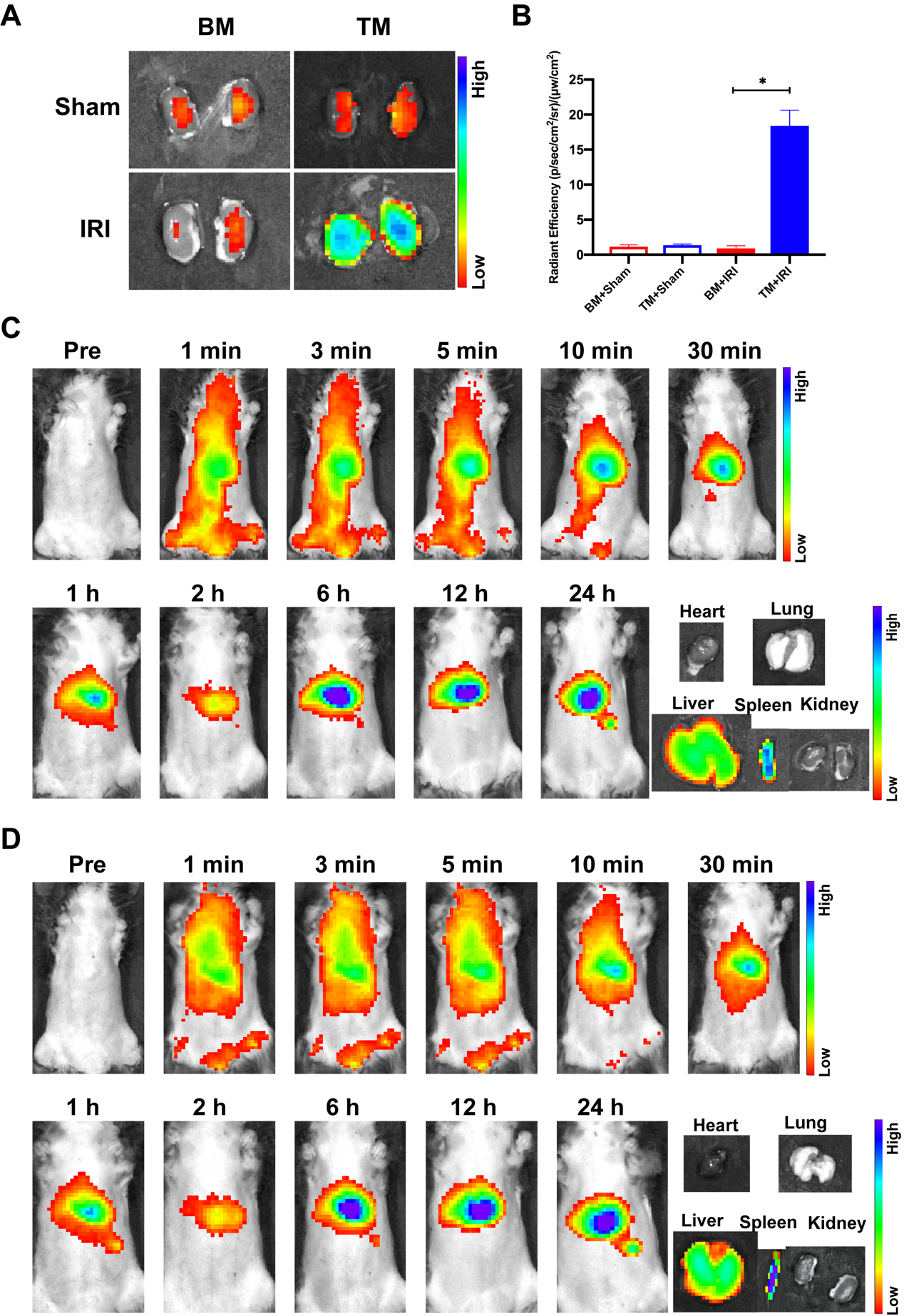


**Supplementary Figure 6. In vivo distribution of microbubbles.** (A) Aggregation of TM and BM in the kidney. (B) Quantitative analysis of kidney fluorescence intensity of TM and BM (n=3 per group). Data in the graph represent the mean ± SD, and p value was determined by one-way ANOVA and Dunnett's T3 post hoc test. Fluorescence images of DiR-labelled (C) BM or (D) TM in vivo distribution. *P < 0.05. BM: blank microbubbles; IRI: Ischemia-reperfusion injury; TM: targeted microbubbles.
